# Proteomic Profiling of Two Distinct Populations of Extracellular Vesicles Isolated from Human Seminal Plasma

**DOI:** 10.3390/ijms21217957

**Published:** 2020-10-26

**Authors:** Xiaogang Zhang, Harmjan R. Vos, Weiyang Tao, Willem Stoorvogel

**Affiliations:** 1 Department of Biomolecular Health Sciences, Faculty of Veterinary Science, Utrecht University, 3584 CM Utrecht, The Netherlands; x.zhang@uu.nl; 2 Center for Molecular Medicine, Molecular Cancer Research Section, University Medical Center, 3584 CX Utrecht, The Netherlands; H.R.Vos-3@umcutrecht.nl; 3 Department of Rheumatology and Clinical Immunology, University Medical Center Utrecht, Utrecht University, 3508 GA Utrecht, Netherlands; W.Tao@umcutrecht.nl

**Keywords:** extracellular vesicles, prostasomes, subtypes, seminal plasma, proteomic analysis, LC-MS/MS

## Abstract

Body fluids contain many populations of extracellular vesicles (EV) that differ in size, cellular origin, molecular composition, and biological activities. EV in seminal plasma are in majority originating from prostate epithelial cells, and hence are also referred to as prostasomes. Nevertheless, EV are also contributed by other accessory sex glands, as well as by the testis and epididymis. In a previous study, we isolated EV from seminal plasma of vasectomized men, thereby excluding contributions from the testis and epididymis, and identified two distinct EV populations with diameters of 50 and 100 nm, respectively. In the current study, we comprehensively analyzed the protein composition of these two EV populations using quantitative Liquid Chromatography-Mass Spectrometry (LC-MS/MS). In total 1558 proteins were identified. Of these, ≈45% was found only in the isolated 100 nm EV, 1% only in the isolated 50 nm EV, and 54% in both 100 nm and 50 nm EV. Gene ontology (GO) enrichment analysis suggest that both originate from the prostate, but with distinct biogenesis pathways. Finally, nine proteins, including KLK3, KLK2, MSMB, NEFH, PSCA, PABPC1, TGM4, ALOX15B, and ANO7, with known prostate specific expression and alternate expression levels in prostate cancer tissue were identified. These data have potential for the discovery of EV associated prostate cancer biomarkers in blood.

## 1. Introduction

Seminal plasma is a complex fluid composed of secretory products from the testis, the epididymis, the prostate, and the seminal vesicles [1,2]. In addition to its function as carrier of sperm cells, seminal plasma provides essential support for the survival and maturation of sperm cells in the female reproductive tract [3]. Seminal plasma contains a high concentration of extracellular vesicles (EV) [4]. Proposedly, seminal plasma EV can regulate multiple sperm cell functions, including sperm motility [5], the acrosome reaction [6], and sperm cell capacitation [7]. In addition, seminal plasma EV may protect sperm cells by antibacterial and antioxidant activities [8,9]. Seminal EV also have immune regulatory capacities that are thought to tolerize maternal immune cells within the female reproductive tract for semen, and in case of successful fertilization also for paternal antigens that are expressed by the fetus [10,11,12,13,14,15]. A potential adverse effect of immune inhibitory properties of seminal EV is that they may facilitate dissemination of pathogens during sexual intercourse, including human immunodeficiency virus and human papillomavirus [14]. Another hypothetical adverse effect of these EV is that they may tolerize the male immune system towards prostate cancer, potentially contributing to the high incidence of this disease [16]. The molecular mechanisms by which seminal EV regulate all these different biological processes have not been fully elucidated. The distinct biological functions of EV subtypes are likely to be reflected by their cargo [17,18,19]. EV from prostate epithelial cells are in majority secreted by multivesicular bodies (MVB) or “storage vesicles” [20,21] and hence can be classified as exosomes [22], although also some larger microvesicles seem to be formed by apocrine secretion [23]. Exosomes are generated as intraluminal vesicles (ILV) of MVB, during which certain cargoes, including protein, lipid, and nuclear acids, are selectively incorporated [24,25,26]. The proteome of isolated EV from seminal plasma has been analyzed by mass spectrometry by multiple research groups (see Table 1 in the results section), but in these studies the EV were isolated from seminal plasma from intact men, which in addition to EV from the prostate also contains EV from the testis and epididymis, including the so-called epididymosomes [27]. Consistent with such distinct sources, EV in seminal plasma are diverse with respect to their size and morphology [28]. Prostate derived EV, without contaminating EV from the testis or epididymis, can be isolated from seminal plasma of vasectomized man [4,29]. We previously identified two distinct EV subtypes from seminal plasma of vasectomized men with characteristic diameters of ≈50 and ≈100 nm, respectively, and distinct protein and lipid compositions [24,30]. In the current study, we comprehensively analyzed the protein content of these two EV subtypes by quantitative LC-MS/MS. GO enrichment analysis is consistent with distinct biological functions and mechanisms for their biogenesis. In addition, several prostate specific proteins with alternate expression levels in prostate cancer tissue were detected in these EV.

## 2. Results

### Isolation of Two Populations of Seminal EV

**Figure 1:**
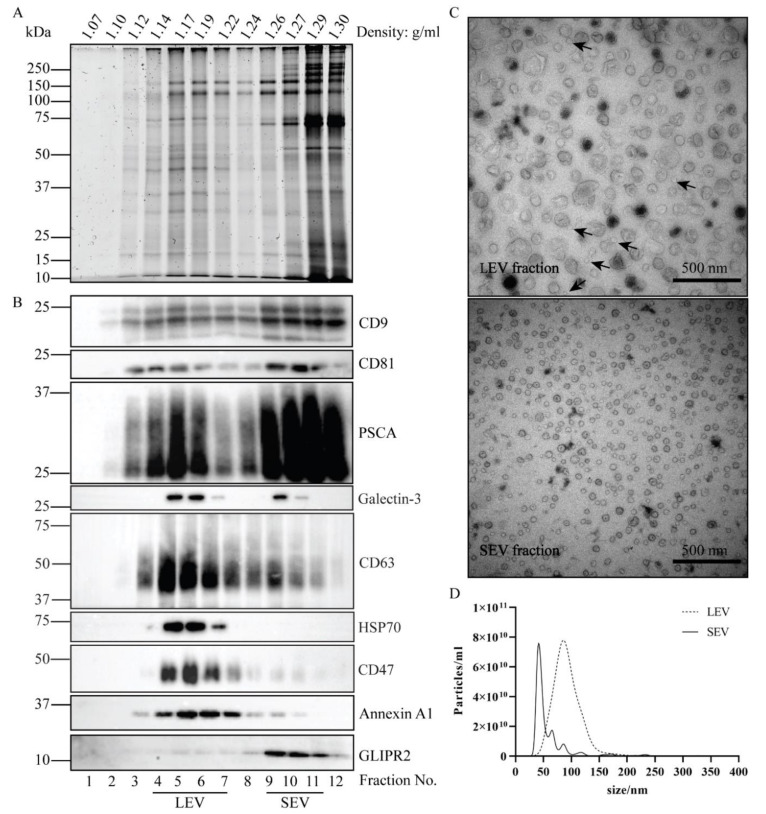
Isolation and characterization of LEV and SEV. Total EV in seminal plasma of vasectomized men were collected by UC at the interface of a sucrose block gradient. LEV and SEV were separated by their distinct velocities during upward displacement into a continuous sucrose density gradient. (**A**) Gradient fractions were analyzed by SDS-PAGE followed by Sypro ruby staining for total protein. (**B**) Gradient fractions were analyzed by immunoblotting for the presence of EV associated proteins, including CD9, CD81, PSCA, Galectin-3, CD63, HSP70, Annexin A1, and GLIPR2. Molecular weight markers are indicated on the left in kDa. (**C**) Particles in the LEV and SEV containing fractions were analyzed by TEM. Scale bar, 500 nm. Arrows exemplify incidental SEV in the LEV isolate. (**D**) Size distribution of the particles in LEV and SEV isolates as determined by NTA.

EV were isolated from pooled batches of seminal plasma, with each pool containing donations from three separate individuals. Seminal samples were obtained from vasectomized men to exclude contributions from the testis or epididymis. The workflow for EV isolation is depicted in Figure S1. After removing large sized particles by a series of centrifugation steps up to 10,000× *g*, EV were centrifuged for 2 h at 100,000× *g* into a sucrose block gradient and collected at the interface of 0.7 M and 2 M sucrose. Soluble proteins retained on top of the 0.7 M sucrose cushion. The EV containing fraction was then adjusted to > 2 M sucrose, loaded at the bottom of a tube, overlaid with a linear sucrose density gradient, and floated upward into the gradient during 16 h centrifugation at 190,000× *g*. The migration velocity of EV in highly viscous sucrose density gradients is dependent on their size, and we have previously demonstrated that a population of ≈100 nm large EV (LEV) reached their equilibrium buoyant density already after 16 h centrifugation. In contrast, a population of ≈50 nm small EV (SEV) required at least 64 h centrifugation to reach their equilibrium buoyant density, which is identical to that of LEV [30]. Based on these distinct velocities, the two populations of EV were separated after 16 h centrifugation. SDS-PAGE followed by total protein staining revealed two EV peaks, represented by fractions 4–7 and fractions 9–11, each with distinct protein patterns (Figure 1A). Immunoblotting demonstrated the shared presence of conventional EV markers in both peaks, including CD9, CD81, CD63, and Galectin-3 (Figure 1B). Both peaks also contained Prostate Stem Cell Antigen (PSCA), a prostate specific marker, indicating the prostate as their shared origin. Annexin A1, HSP70, and CD47 were relatively much more dominant in fractions 4–7, while GLIPR2 was almost exclusively found in fractions 9–11, consistent with distinct protein compositions of the two EV populations (Figure 1B). To collect LEV and SEV separately, fractions 4–7 and fractions 9–11 were pooled, diluted with PBS, and ultracentrifuged (Figure S1). Analysis by TEM indicated diameters of isolated LEV and SEV of ≈100 nm versus ≈50 nm, respectively (Figure 1C), consistent with the average diameters of 92.5 ± 1.0 and 59.4 ± 2.2 nm that were measured by NTA (Figure 1D). It should be noted that, although no LEV could be discerned in the SEV fraction, some contaminating SEV were detected by TEM in the LEV fraction (indicated by arrows in Figure 1C). Possibly, the migration velocity in the density gradient of the contaminating SEV in the LEV containing gradient fractions was increased as a consequence of clustering of SEV with other SEV or LEV. To illustrate reproducibility of the SEV and LEV isolation procedure, a second, independently performed duplicate experiment is shown in Figure S2.

**Figure 2:**
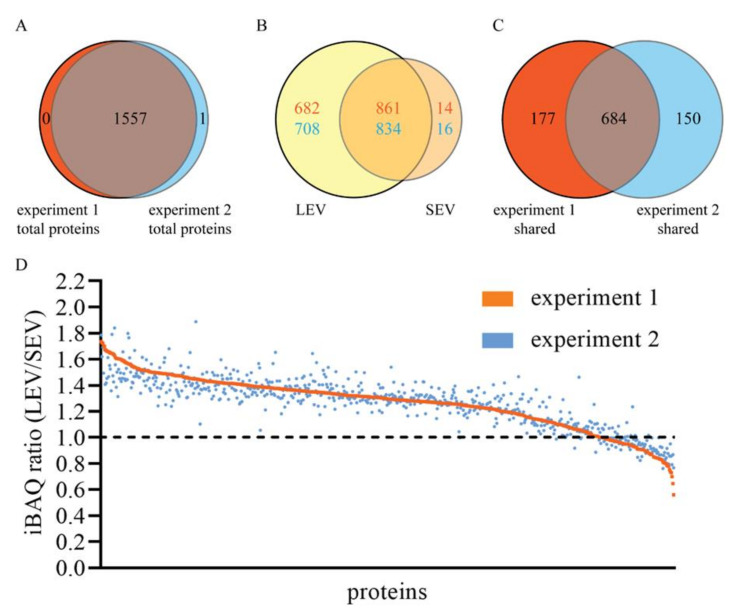
MS/MS identification and relative quantification of proteins in LEV and SEV. (**A**) Venn diagram indicating the total number and overlap of proteins in LEV and SEV detected in experiment 1 (red) and experiment 2 (blue). (**B**) Venn diagram comparing of proteins detected in LEV (yellow) and SEV (beige) in experiment 1 (red numbers) and experiment 2 (blue numbers). (**C**) Venn diagram comparing proteins that are shared by LEV and SEV in experiment 1 (red) with those in experiment 2 (blue). (**D**) iBAQ ratios (LEV/SEV) for all 684 proteins that shared within both experiment 1 and experiment 2, as indicated in C. Proteins are plotted on x-axes by decreasing iBAQ ratio in experiment 1 (red line). The same proteins are plotted in the same order along the x-axes for experiment 2 (blue dots).

**Figure 3:**
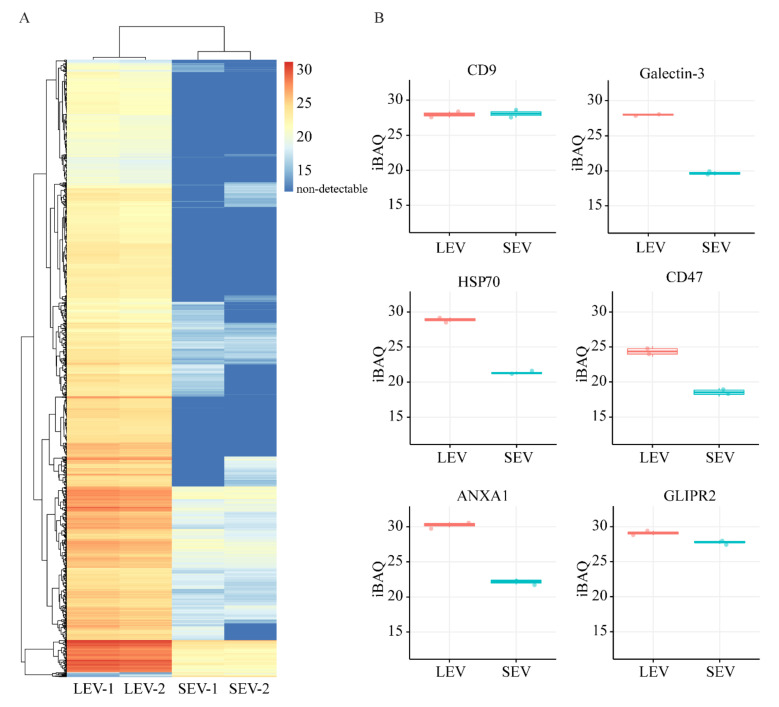
(**A**) IBAQ heatmap for all 1557 proteins that were detected either in LEV and/or SEV in both experiments (see Figure 1A). Color code key on the right indicates iBAQ values. Red color is corresponding to relatively high abundance of proteins, darkest blue indicates absence of protein. (**B**) IBAQ values of six selected proteins in LEV and SEV that were also compared by immunoblotting (see Figure S3B).

The protein compositions of the isolated LEV and SEV were comprehensively analyzed by quantitative LC-MS/MS. In total, 1558 proteins were identified in two independently performed experiments (Table S1), with only one protein remaining undetected in one of the two experiments, demonstrating high reproducibility (Figure 2A). The protein compositions of LEV and SEV were very different, with 861–834 proteins detected in both LEV and SEV isolates, 682–708 proteins only in LEV, and 14–16 proteins only in SEV (Figure 2B). Thus, in both experiments, >98% of all proteins found in the highly purified SEV could also be detected in the LEV isolate. In contrast, ≈45% of all proteins that were found LEV could not be detected in SEV. The overlap in protein profiles could indicate sharing of these proteins between LEV and SEV, but alternatively may be explained by contamination of the LEV isolated with some SEV, as suggested by TEM analyses (Figure 1C). To distinguish between these two possibilities, we compared the relative quantity (LEV versus SEV) of all 684 proteins that were shared by LEV and SEV in both experiments, using the iBAQ algorithm (Table S1 and Figure 2C and Figure 3A). For validation, we compared this approach with immunoblotting. Of the seven proteins for which relative distributions over LEV versus SEV were determined by immunoblotting (Figure S3), five proteins showed comparable distributions according to their relative iBAQ values (Figure 3B). GLIPR2 is an exception, however, as it was enriched in the SEV fraction as determined by immunoblotting, but not according to its iBAQ ratio. Despite a clear immunoblot signal, CD63 was not picked up by LC-MS/MS, exemplifying that membrane bound proteins often remain undetected by the latter approach, especially when heavily glycosylated. Of all 684 proteins that were shared by LEV and SEV, iBAQ ratios for LEV versus SEV ranged between 1.9 and 0.6, with an average of 1.3 ± 0.2 (mean ± S.D.), indicating that the great majority of these proteins is truly shared by LEV and SEV. Moreover, the variation in iBAQ ratios between the two experiments for individual proteins was remarkably small (Figure 2D), again indicating reproducibility of the data.

**Figure 4:**
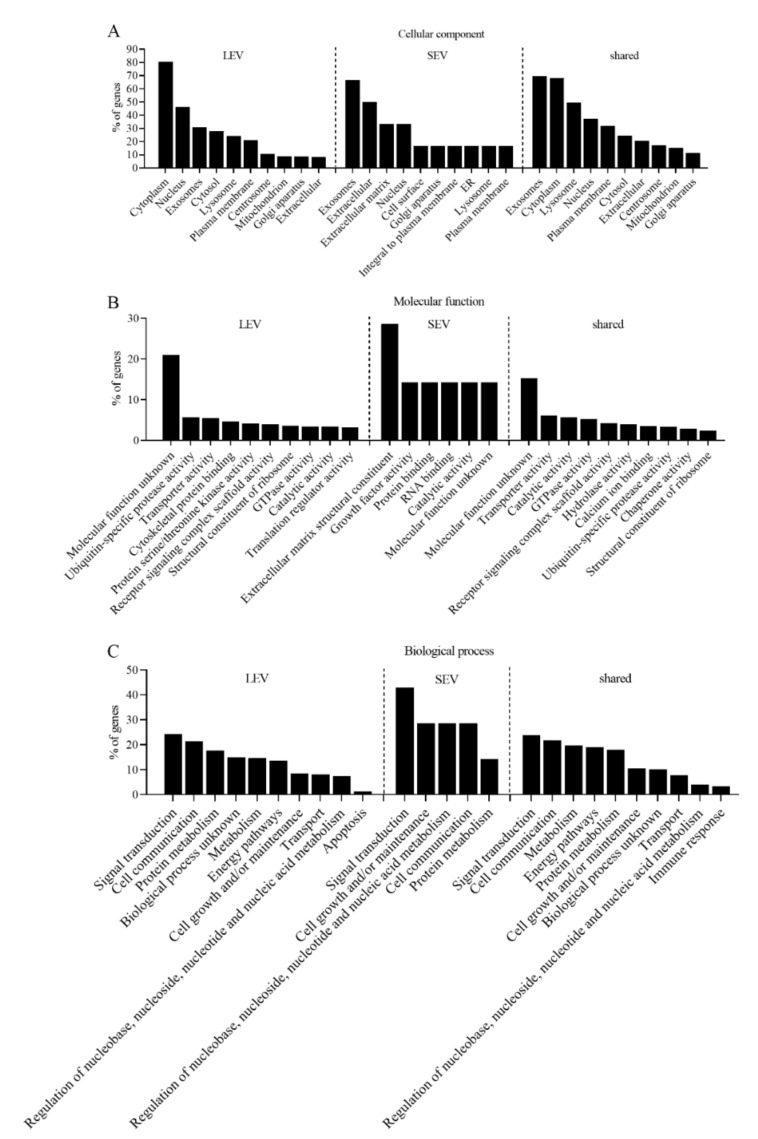
Gene ontology (GO) annotations of proteins detected in both experiments in LEV only (539 proteins), SEV only (seven proteins), or shared by LEV and SEV (684 proteins). Ranking is according to the database in FunRich and ordered by the percentage of genes per GO term. (**A**) Cellular component annotations. (**B**) Molecular function annotations. (**C**) Biological process annotations.

GO enrichment analysis was performed to classify the 684 proteins that were shared by LEV and SEV in both experiments, the 539 proteins that were exclusively detected in LEV, and the seven proteins exclusively detected in SEV (Figure 4). Consistent with an exosomal identity, the cellular component “exosomes” is prominent in all three classes. As much as 21% proteins in LEV and 15% of the proteins shared by LEV and SEV have unknown molecular functions. Molecular functions of many other LEV associated proteins are classified as “ubiquitin-specific protease activity”, “transporter activity”, or “cytoskeletal protein binding”. Comparable biological process GO terms distributions were found for LEV and LEV/SEV shared proteins, and “signal transduction” is prominent for both classes, consistent with a role of both LEV and SEV in intercellular communication.

**Figure 5:**
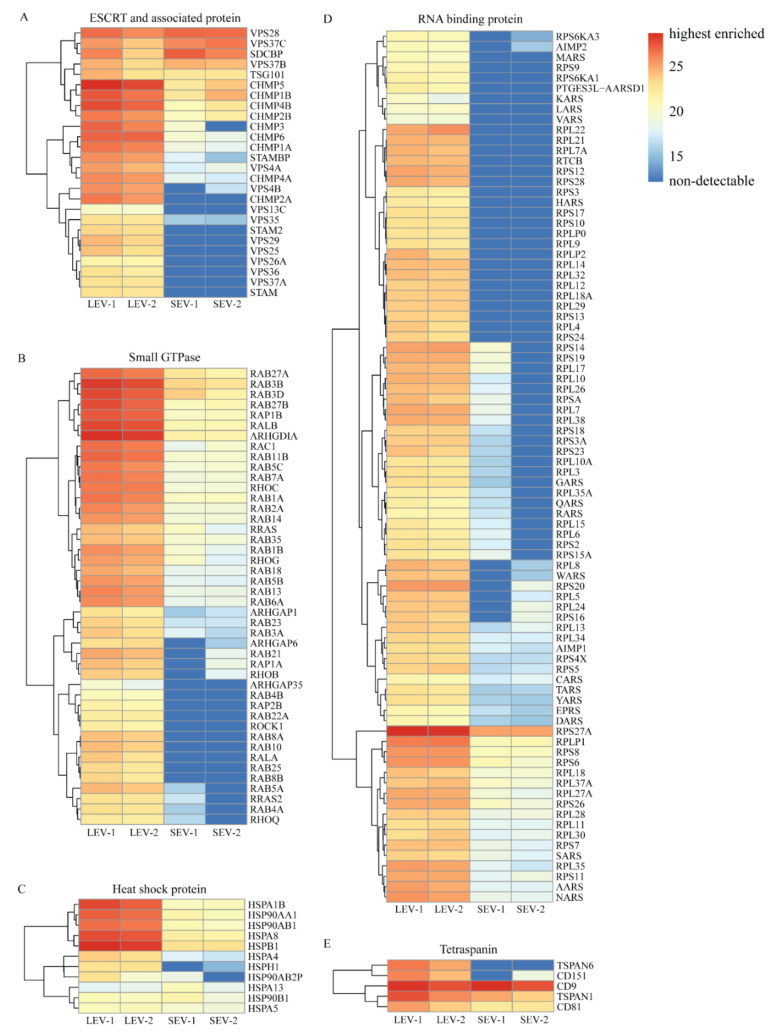
IBAQ heatmaps for specific protein groups potentially related to EV biogenesis. (**A**) ESCRT and associated proteins. (**B**) Small GTPases. (**C**) Heat shock proteins. (**D**) RNA binding proteins. (**E**) Tetraspanin proteins. Color code key on the right indicates iBAQ values. Red color is corresponding to relatively high abundance of proteins, darkest blue indicates absence of protein.

Proteins that are implicated in EV biogenesis or cargo loading, including endosomal sorting complexes required for transport (ESCRT), small GTPases, heat shock proteins, RNA binding proteins, and tetraspanins are abundantly represented (see heatmap in Figure 5). Among the ESCRT and ESCRT-associated proteins, only VPS28, VPS37C, and SDCBP were relatively more abundant in SEV isolates, while all others were either relatively enriched in LEV or even completely absent in SEV isolates. All small GTPases, heat shock proteins, and RNA binding related proteins were either more enriched in LEV as compared to SEV isolates, or not at all detected in SEV. Within the category of tetraspanins, TSPAN6 and CD151 were detected in LEV but not in SEV, while CD9, TSPAN1, and CD81 were present in both LEV and SEV (Figure 5E).

**Figure 6:**
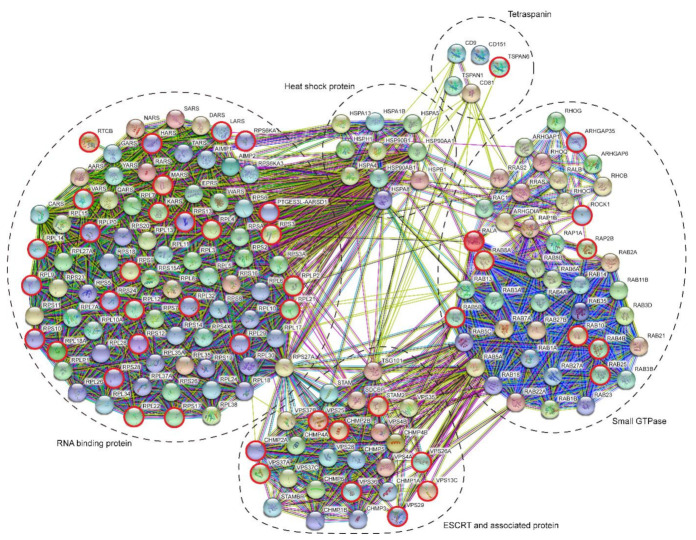
Protein–protein interaction network as determined by STRING for all proteins shown in Figure 5. Red encircled proteins were detected in LEV only. All other proteins were detected in both LEV and SEV. Dotted lines indicate the different protein groups in Figure 5A–E. Note that these protein groups are maintained by this presentation. Additionally, note the linkage between “RNA binding proteins” and “ESCRT and associated proteins” by RPS27A, and that both “tetraspanins” and “small GTPases” are linked to “RNA binding proteins” via “heat shock proteins”.

A protein–protein interaction network (STRING) of all ESCRT associated proteins, small GTPases, heat shock proteins, tetraspanins, and RNA binding proteins is shown in Figure 6. Interestingly, according to this analysis, these five classes of proteins come back as separate but interconnected clusters. The ribosome associated protein RPS27A is particularly interesting in this respect as it forms a major link between the clusters of RNA binding proteins and ESCRT associated proteins (Figure 6) and is quantitatively very prominently present in LEV as suggested by the highest iBAQ value within the group of RNA associated proteins (Figure 5D).

## 3. Discussion

In a previous study, we identified LEV and SEV as two separate classes of EV in the seminal plasma of vasectomized men [30]. In the current study, we performed a comprehensive analysis of the proteome of LEV and SEV and identified a total of 1558 LEV/SEV associated proteins. We established that the protein compositions of LEV and SEV are distinct but partly overlapping. The size and molecular composition of LEV and SEV suggests that both are generated as exosomes from prostate epithelial cells, but by distinct molecular machineries, and that they have distinct biological functions.

### 3.1. The Origin of LEV and SEV

Comparison of the proteome of LEV and SEV with previously published data from other laboratories (Table 1) indicates overlapping protein profiles as well as large differences. These differences may be related to differences in the source of seminal plasma (vasectomized versus non-vasectomized), methodologies used for EV isolation, and/or sensitivities of protein detection.

Consistent with EV heterogeneity, several subtypes of EV were also found in seminal plasma from non-vasectomized men [28,31]. It should be kept in mind, however, that seminal plasma from non-vasectomized men, but not from vasectomized men, also contains epididymosomes that are released by the epididymis, and membrane fragments from dying sperm cells. Although contributions from the testis or epididymis can be excluded in our study on seminal plasma EV from vasectomized men, seminal vesicles (also called seminal glands or vesicular glands) are likely to have contributed. EV have indeed been found in fluid aspirated directly from operationally dissected seminal vesicles [35]. Contributions from seminal vesicles were difficult to determine in our EV isolates, since no proteins are known to be exclusively expressed by seminal vesicles, as compared to the prostate, according to the human protein atlas [36]. Many proteins that are abundantly secreted by seminal vesicles are also expressed by the prostate, including semenogelin-1 and semenogelin-2. We found semenogelin-1 and -2 in association with both LEV and SEV with IBAQ ratios (LEV/SEV) of ≈0.9, indicating that these secretory proteins associate with similar extent to the two EV subtypes. According to the human protein atlas [36], 180 mRNA transcripts are expressed more abundantly in seminal vesicles relative to other tissues. Within this pool, we found six translation products that were detected by immunohistochemistry in seminal vesicles but not in the prostate [36], as well as by us in LEV and SEV using LC-MS/MS. These proteins include antileukoproteinase, prostaglandin E synthase 3, aldose reductase, prolactin-inducible protein, ectonucleotide pyrophosphatase, and Gamma-glutamyltranspeptidase 1. Except for antileukoproteinase, these proteins are either cytosolic or associated with membrane. Together, these data indicate that some LEV and SEV in seminal plasma derive from seminal vesicles.

Of the 14,928 genes expressed in prostate tissue, only 120 are highly restricted to the prostate as compared to other tissues [36]. Of these prostate-specific proteins, we found nine in LEV/SEV (see below for their identity), confirming that many LEV and SEV truly represent prostasomes. Consistent with the idea that LEV and SEV can be generated as exosomes by prostate epithelial cells, electron microscopic studies of the prostate revealed the abundant presence of small 50–100 nm sized EV in the prostate acini as well as in the lumen of MVB like structures within prostate epithelial cells [20,23]. However, the latter study also reported larger microvesicle type EV, with diameters ranging from 150 nm up to several µm, that appear to pinch off from the apical plasma membrane, which would classify these EV as microvesicles. Such relatively large EV have also been detected, although in a very limited extent in the ejaculate of non-vasectomized men [28] and in seminal plasma from vasectomized men [30]. Importantly, in our current study, such large EV have been depleted from the seminal plasma by 10,000× *g* centrifugation.

Prostasomes have been proposed to regulate sperm cell capacitation, the acrosome reaction, sperm motility, and immune tolerance within the female reproductive tract (reviewed by Aalberts [4]) [14]. It is plausible that such different functions are performed by distinct EV subpopulations. Indeed, our GO enrichment analysis of the proteome of isolated LEV and SEV suggests distinct molecular functions (Figure 4A) and biological processes (Figure 4B) for these two classes. It would go too far here to speculate on all potential functions of LEV and SEV in relation to the proteins identified therein. Nevertheless, this proteome analysis may provide hints to investigate potential molecular players in prostasome functions. For example, CD47 is highly enriched in LEV relative to SEV, as determined both by LC-MS/MS (Table S1) and immunoblotting (Figure 1A and Figure S3). CD47 is a ubiquitously expressed glycoprotein of the immunoglobulin superfamily that is overexpressed in nearly all types of tumors, and acts as a do not eat me signal to macrophages of the immune system [33,37]. Sperm cells may escape phagocytosis by macrophages by acquiring CD47 from LEV, similar to the CD47-dependent immunomodulatory function of EV from T cells [38]. A potential role of CD47 in immunomodulation is just one example of how the role of prostasomes in immune regulation can be investigated in follow-up studies.

### 3.2. Indications for Mechanisms of LEV/SEV Formation and Cargo Loading

Although the molecular mechanisms that drive EV formation and the incorporation of cargo therein are not entirely resolved, it can be expected that separate mechanisms are required for the formation of LEV and SEV and their incorporation of different sets of proteins. Both LEV and SEV correspond in size to the intraluminal vesicles detected by electron microscopy in storage vacuoles (the equivalent of MVB) within prostate epithelial cells, rather than with much larger and less numerous protrusions detected at their plasma membrane [39]. This alone would already classify both LEV and SEV as exosomes. Exosomes from other cell types also vary in size from 40 to 100 nm, consistent with the variation in size of intraluminal vesicles of MVB [40]. Parallel molecular mechanisms are involved in exosome formation, including the ESCRT machinery. ESCRT is composed of ESCRT-0, ESCRT-I, ESCRT-II, and ESCRT-III subcomplexes, and a number of accessory proteins. ESCRT-0 is responsible for clustering of ubiquitinated cargo proteins, while ESCRT-I and ESCRT-II induce inward budding of the MVB delimiting membrane, and ESCRT-III drives vesicle scission [41]. We detected the ESCRT-I components VPS23 (TSG101), VPS28, and VPS37 isoforms, as well as the ESCRT-III subunits (CHMP1 to 6) in both LEV and SEV. Interesting though, the ESCRT-0 subunits STAM1 and STEM2, as well as the ESCRT-II subunits VPS25 and VPS36 were detected in LEV only. These data suggest that different molecular mechanisms are involved in the formation of LEV and SEV. Sorting of many membrane proteins into ILV of MVB relies on their ubiquitination, driving their recruitment by ESCRT-0. After recruitment, however, cargo proteins are deubiquitinated by deubiquitinating enzymes before ILV are pinched off from the MVB delimiting membrane. Interestingly, proteins with ubiquitin-specific protease activity form the second largest group of LEV specific proteins according to the GO enrichment analysis for molecular function (Figure 4). Combined these data suggest that cargo recruitment into LEV, but not into SEV, may rely on transient ubiquitination. Indeed cargo recruitment into exosomes can also occur independently of ESCRT, and MVB are still formed in cells that are depleted of key ESCRT components [42]. Several ESCRT independent mechanisms for recruitment of cargo into exosomes have been proposed, including a role for the tetraspanin family [22,43,44]. We detected the tetraspanins CD9, CD81, CD63, TSPAN 1, CD151 in both LEV and SEV, by using LC-MS/MS and/or immunoblotting, while TSPAN6 was found exclusively in LEV. Syntenin is another protein with a capacity to recruit cargo proteins into exosomes independently of ubiquitination, and this pathway relies on the small GTPase ARF6 and ALIX [45]. Although we found syntenin in both LEV and SEV, both ARF6 and ALIX were not detected, suggesting that this pathway may not play an important role here. In conclusion, analysis of the protein composition of LEV and SEV suggest that recruitment of their cargos may depend on different mechanisms, with SEV being less dependent on the recruitment of ubiquitinated proteins.

EV from many sources have been reported to contain RNA [46,47]. Additionally, EV from human seminal plasma were reported to contain many types of RNA, including miRNA, tRNA, and Y RNA [26]. We observed that RNA in seminal plasma EV was largely restricted to LEV (our unpublished data). Consistent with these observations, RNA associated proteins were also largely restricted to LEV. The sorting of RNA into EV is regulated by proteins [48]. For example, KRAS is involved in sorting of miRNA into exosomes [49], while YBX1, plays crucial roles in the sorting of Y RNA and tRNA (fragments) into exosomes [50]. KRAS and YBX1 were highly enriched in LEV (Figure 5D). RPS27A is translated as a linear fusion with ubiquitin, which is cleaved off before the ribosome reaches translation competence [51], but, like many ubiquitinated proteins, may also associate with the ESCRT complex. In this way its ubiquitin moiety may drive its sorting into EV and help incorporation of 40S ribosomal subunit associated tRNA and other RNA species into prostasomes.

### 3.3. Potential Discovery of Prostasome Associated Biomarkers for Prostate Cancer

To identify prostate-specific proteins in LEV and SEV, we compared their proteome with the prostate specific proteome in the human protein atlas [36] and found nine hits: kallikrein related peptidase 3, kallikrein related peptidase 2, microseminoprotein beta, neurofilament heavy chain, prostatic acid phosphatase, polyadenylate-binding protein 1, transglutaminase 4, 15-Lipoxygenase-2, and anoctomin-7. Interestingly, all these nine prostate-specific proteins that we found in LEV and SEV are linked to prostate cancer. Kallikrein related peptidase 3 (KLK3), also referred to as prostate specific antigen (PSA), is a well-known biomarker in blood for prostate cancer [52,53], secreted by prostate epithelial cells. PSA normally functions in hydrolyzing seminogelin-1 and -2, which are largely contributed by the seminal vesicles, thereby liquefying the seminal coagulum. Others also found PSA on prostasomes, possibly in association with Galectin-3 [54]. Similar to KLK3, also Kallikrein related peptidase 2 (KLK2) functions in hydrolyzing seminogelins and is regulated by androgen receptor [55], upregulated in prostate tumor cells, and recognized as potential prognostic maker for prostate cancer risk [56]. Microseminoprotein beta (MSMB) is a member of the immunoglobulin binding factor family which is highly expressed and secreted by prostate epithelial cells. MSMB has inhibin-like activity, inhibits growth of cancer cell lines, and was often found reduced or even lost in prostate cancer tissue [57]. Neurofilament heavy chain (NEFH) is a protein that is, in addition to the central nervous system and in peripheral nerves, also expressed by normal prostate epithelial cells but downregulated in prostatic carcinomas [58]. Prostatic acid phosphatase (PAP), also named prostatic specific acid phosphatase (PSAP), is encoded by ACPP, secreted by the prostate under androgen regulation, found in high levels in metastasized prostate cancer, and used as marker in immunohistology for prostate cancer [59]. In seminal plasma, PSAP inactivates lysophosphatidic acid and converts AMP to adenosine. Polyadenylate-binding protein 1 (PABPC1) expression is associated with prostate cancer [60]. PABPC1 is a cytosolic protein that binds, chaperones, and regulates mRNA functions. It is attractive to suggest that PABPC1 may facilitate RNA loading of extracellular vesicles, but this is entirely speculative at this time. Transglutaminase 4 (TGM4) is often highly expressed in prostate cancer and its expression levels in EV isolated from urine were strongly associated with prostate cancer [61]. 15-Lipoxygenase-2 (ALOX15B) is an arachidonic acid metabolizing enzyme that has been implicated as a functional tumor suppressor for prostate cancer, but also other cancers [62]. Anoctomin-7 (ANO7) is a member of the anoctamin family of Ca^2+^ activated Cl^−^ channels, highly expressed by the prostate, and associated with aggressive prostate cancer [63].

According to the pathology atlas of the human cancer transcriptome [64], 37 genes are overexpressed in prostate cancer only (as compared to other cancer types). Two out of these 37 of these gene products, semenogelin 2 (SEMG2) and TGM4, were also present in our LEV and SEV isolates. Similar to healthy cells, prostate epithelial cancer cells produce EV that can be isolated from seminal fluid and urine. In healthy tissue, prostasomes are released into the prostatic ductal system as a consequence of fusion of MVB/storage vesicles with the apical plasma membrane of the epithelial cell. Prostate cancer is characterized by loss of polarity of epithelial cells and the integrity of the basal lamina, allowing the release of prostasomes into the interstitial space and into circulation [16]. Prostasomes in blood may thus serve as a potential biomarker for prostate cancer, indiscriminately of overexpression therein of prostate cancer specific proteins. Detection of a single prostasome specific protein, even when ubiquitously expressed, in a total EV fraction isolated from blood may be sufficient to detect prostate cancer, as exemplified by a study in which the antiapoptotic protein survivin was found to be significantly increased in EV isolated from the plasma of prostate cancer patients compared to patients with proinflammatory benign prostate hyperplasia or healthy controls [65]. However, survivin is also incorporated in EV from other types of cancer [66,67] and thus not prostate cancer specific. Our identification of SEMG2 and TGM2 on LEV/SEV, two protein that are expressed by multiple tissues but overexpressed only in prostate cancer, and nine other prostate specific proteins that are associated with LEV/SEV and also have an altered expression in prostate cancer, is an excellent lead to the identification of prostate cancer-specific EV-associated markers in blood. More studies are required to investigate the potential of these proteins as EV associated prostate cancer biomarker in blood. Only recently we reported a novel three step protocol to isolate extracellular vesicles from blood plasma with both high yield and purity [68], allowing the testing of EV associated prostate cancer biomarker in blood with maximal signal to noise ratio.

## 4. Materials and Methods

### 4.1. Isolation of LEV and SEV from Seminal Plasma from Vasectomized Men

Human semen was collected at the Meander Medical Center (Amersfoort, the Netherlands) from healthy vasectomized man following a 3 month postoperative period. Informed consent was obtained, and the investigation approved by the Medical Ethics Committee at Meander Medical Center (permission code TWO-11-62, 2 December 2011). Successful vasectomy was confirmed by lack of sperm cells as determined by phase contrast microscopy Olympus BX40, Hamburg, GermanySeminal plasma samples were cooled to 4 °C and this temperature was maintained during the entire EV isolation procedure. For each experiment, EV were isolated from pooled seminal plasma samples of three different donors. EV isolation was principally performed as described previously [30], with minor modifications. The pooled seminal plasma samples were diluted with an equal volume of PBS and centrifuged for 10 min at 3000× *g* at 4 °C to remove any debris. The supernatants were collected and centrifuged for 30 min at 10,000× *g* at 4 °C to remove large vesicles (large microvesicles and apoptotic bodies). The supernatants were collected and passed through a filter with 0.2 µm pore size to remove any remaining large particles or pathogens. The filtrate was carefully layered on top of 3 mL 0.7 M sucrose, 100 mM NaCl, 20 mM HEPES/NaOH pH 7.4, which on its turn had been layered on top of 2 M sucrose, 100 mM NaCl, 20 mM HEPES/NaOH pH 7.4 in a SW28 tube. The block gradients were then centrifuged for 2 h at 100,000× *g* at 4 °C in a SW28 rotor (Beckman Instruments, Brea, California, USA). The EV-containing fraction of ≈2 mL was collected from the interface between the two sucrose layers. Solid sucrose was dissolved into the collected fraction to reach a > 2 M sucrose concentration, as determined by refractive index measurements. The samples were then loaded in SW40 tubes and overlaid with linear sucrose density gradients (2.0 M-0.4 M sucrose in 100 mM NaCl, 20 mM HEPES/NaOH pH 7.4). The EV were floated upward into the gradients during 16 h centrifugation at 190,000× *g*. Consecutive fractions of 1 mL (fraction 1–11) were collected from the top, with a 12th bottom fraction of ≈2 mL. Fraction densities were determined by refractometry. LEV containing fractions were identified by immunoblotting for the presence of HSP70, AnnexinA1 and CD47, SEV by the presence of GLIPR2 (see below). LEV and SEV containing fractions were pooled separately, diluted with PBS, and centrifuged for 2 h at 100,000× *g*. Pelleted EV were resuspended in PBS and recentrifuged. Washed EV were resuspended in PBS. Protein concentrations were determined using BCA protein assay (TD265235, Thermo Fisher Scientific, Rockford, Illinois, USA) according to the manufacturer’s instructions. Isolated SEV and LEV were frozen at −80 °C until further use.

### 4.2. SDS-PAGE and Immune-Blotting

Gradient fractions were analyzed by 10% SDS polyacrylamide gel electrophoresis (SDS-PAGE) followed by total protein staining using Sypro ruby (1890270, Invitrogen, Eugene, Oregon, USA) according to the manufacturer’s procedure. Sypro ruby fluorescently labelled proteins were detected using a Bio-Rad ChemiDoc imaging system (Bio-Rad, Hercules, California, USA). For immune-blotting, proteins were separated by 10% SDS-PAGE and transferred to PVDF membranes (Millipore, Billerica, USA). Prestained protein marker (Bio-Rad, Hercules, California, USA) was used as a molecular weight standard. The blots were blocked for 1 h at room temperature in PBS containing 0.1% Tween-20 and 0.5% fish skin gelatin, and incubated overnight in the same buffer at 4 °C with primary antibodies. The primary antibodies used were mouse anti human CD9 (HI9a, 312102, Biolegend, San Diego, California, USA, 1:2000); mouse anti human CD81 (B11, sc-166029, Santa Cruz Biotechnology, Dallas, Texas, USA, 1:500); mouse anti human HSP70 (N27F3-4, ADI-SPA-820-D, Enzo, Bruxelles, Belgium, 1:1000); mouse anti human Flotillin-1 (clone 18, 610821, BD Biosciences, San Jose, California, USA, 1:1000); mouse anti-human PSCA (clone 7F5, sc-80654, Santa Cruz Biotechnology, Dallas, Texas, USA, 1:1000); mouse anti human Annexin A1 (clone 29, 610066, BD Biosciences, San Jose, California, USA, 1:1000); mouse anti human CD47 (B6H12, sc-12730, Santa Cruz Biotechnology, Dallas, Texas, USA, 1:500); rat anti human galectin-3 (M3/38, CL8942B, CEDARLANE, Burlington, Vermont, USA, 1:500) and rabbit anti human GLIPR2/GAPR-1 (Eberle et al., 2002, 1:5000, kindly provided by J.B. Helms). Primary antibodies were labelled with goat anti mouse HRP (Jackson immune Research, West Grove, Pennsylvania, USA, 1:10,000), goat anti rat HRP (Dako, Santa Clara, California, USA, 1:5000), or goat anti rabbit HRP (Dako, Santa Clara, California, USA, 1:5000). HRP labelled antibodies were detected with Super Signal West Dura Chemiluminescent Substrate (TF267375, Thermo Fisher Scientific, Rockford, Illinois, USA) and a Bio-Rad Chemidoc imaging system (Bio-Rad, Hercules, California, USA).

### 4.3. Transmission Electron Microscopy

For transmission electron microscopy (TEM), 20 µL samples were placed as droplets on parafilm and covered with formvar and carbon coated 150-mesh grids, and incubated for 10 min at room temperature to allow EV binding. Then, the grids were transferred onto a drop of 2% paraformaldehyde in 0.1 M phosphate buffer. After 5 min fixation, the grids were washed six times with milliQ water. For negative staining, the grids were subsequently transferred for 5 min onto droplets containing 0.4% uranyl acetate and 1.8% methylcellulose. Excess fluid was removed using filter paper and the grids were air dried. Samples were imaged using a transmission electron microscope (Fei, Tecnai-12, Eindhoven, the Netherlands) operated at 80 kV.

### 4.4. Nanoparticles Tracking Analysis (NTA)

The size distribution and concentration of particles in LEV or SEV containing fractions were determined by NTA using a NS500 model (Nanosight Technology, Malvern, UK), configured with a high sensitivity sCMOS camera. The concentrated LEV or SEV were diluted to obtain < 200 particles/frame. The camera level was set at 14 and the detection threshold was set at 3. For each sample triplicate videos of 30 s were recorded and analyzed by NTA 3.1 software (Malvern Panalytical, Malvern, UK).

### 4.5. Mass Spectrometry

Samples of 20 µg isolated LEV and SEV were denatured and alkylated in 50 µL 8 M Urea, 1 M ammonium bicarbonate containing 10 mM tris (2-carboxyethyl) phosphine hydrochloride, and 40 mM 2-chloro-acetamide. After 4-fold further dilution with 1 M ammonium bicarbonate and digestion with trypsin (250 ng/200 µL), peptides were desalted with homemade C-18 stage tips (3 M, St Paul, MN, USA). Peptides were eluted with 80% ACN and, after evaporation of the solvent in the speedvac, redissolved in buffer A (0.1% formic acid). After separation on a 30 cm pico-tip column (75 µm ID, New Objective) in-house packed with C-18 material (1.9 µm aquapur gold, dr. Maisch) using a 140 min gradient (7–80% ACN, 0.1% FA), delivered by an easy-nLC 1000 (Thermo, Rockford, Illinois, USA), peptides were electro-sprayed directly into a Orbitrap Fusion Tribrid Mass Spectrometer (Thermo Scientific, Rockford, Illinois, USA). The MS was set in DDA mode with a cycle time of 1 s, in which the full scan (400–1500 mass range) was performed at a resolution of 240,000. Ions reaching an intensity threshold of 15,000 ions were isolated by the quadrupole and fragmented with a HCD collision energy of 30%, using all available parallelizable time. The maximum injection time of the ion trap was set to 50 milliseconds. For identification, Maxquant software (1.6.3.4, Max Planck Institute of Biochemistry, Munich, Germany) used the Human Uniprot database (January 2019) to analyze the raw data, with oxidation and protein N-terminus acetylation set as variable and carbamidomethylation of cysteine set as fixed modification. Peptide and protein false discovery rates were set to 1%. The data are deposited at the ProteomeXchange Consortium via the PRIDE partner repository with the dataset identifier PXD021192.

### 4.6. GO Annotation and STRING Analysis

Proteins that were identified by LC-MS/MS with at least three peptides, including at least two Razor and/or unique peptides, were analyzed by FunRich (release March 2017), using the human FunRich protein database as background. GO enrichment analysis was performed on the annotated proteins for cellular component, molecular function, and biological process. Protein–protein interaction among protein groups was analyzed by STRING (version 11.0) (www.string-db.org) [69]. Statistical analysis and graphs were performed using Prism GraphPad 8.0 software (GraphPad Software, San Diego, California, USA).

## Figures and Tables

**Table 1:** Comparison of proteins detected large EV (LEV) and/or small EV (SEV) with previous MS/MS studies on the proteome of isolated human seminal plasma EV. Methods used include ultracentrifugation (UC) and size exclusion chromatography (SEC).

Reference	Isolation Method	Subfractionation	Number of Identified Proteins	Number (%) of Proteins Shared with Our Study
Utleg et al. [25]	UC + SEC	No	139	15 (0.96%)
Poliakov et al. [31]	UC + sucrose block gradient	No	440	315 (20.22%)
Dubois et al. [32]	UC + SEC + sucrose density gradient	Triton-X100 extracted lipid raft associated proteins	377	265 (20.49%)
Yang et al. [33]	UC on sucrose cushion	No	1474	949 (60.91%)
García-Rodríguez et al. [34]	UC	Normozoospermic versus non-normozoospermic men	1282	Protein IDs not available online
Our current study	From vasectomized men. UC into sucrose block gradient + upward displacement into velocity sucrose gradient	LEV and SEV separated by velocity sucrose gradient centrifugation	1558	

## Supplementary Materials

The following are available online at https://www.mdpi.com/1422-0067/21/21/7957/s1, Figure S1: Schematic overview of the method used to isolate LEV and SEV. Figure S2: Independent replicate of experiment in Figure 2. Figure S3: Characterization of the LEV and SEV pooled sucrose density gradient fractions. Table S1: Proteins in LEV and SEV isolates detected by LC-MS/MS.

## Abbreviations

EV	extracellular vesicles
NTA LC-MS/MS	nano-particle tracking analysis liquid chromatography-mass spectrometry
MVB	multivesicular bodies
ILV	intraluminal vesicles
LEV	large extracellular vesicles
SEV	small extracellular vesicles
PSCA	prostate stem cell antigen
GO	gene ontology
ESCRT	endosomal sorting complexes required for transport
KLK3	kallikrein related peptidase 3
PSA	prostate specific antigen
KLK2	kallikrein related peptidase 2
MSMB	microseminoprotein beta
NEFH	neurofilament heavy chain
PAP	prostatic acid phosphatase
PSAP	prostatic specific acid phosphatase
PABPC1	polyadenylate-binding protein 1
TGM4	transglutaminase 4
ALOX15B	15-lipoxygenase-2
ANO7	anoctomin-7
SEMG2	semenogelin 2
