# Supplementary material for: Proteomic Profiling of Two Distinct Populations of Extracellular Vesicles Isolated from Human Seminal Plasma
Source: Int J Mol Sci. 2020 Oct 26;21(21):7957. doi: 10.3390/ijms21217957 (PMC7663558; doi:10.3390/ijms21217957)
Supplement: Supplementary file 1 [file ijms-21-07957-s001.zip › ijms-935540-supplementary figures.pdf]

Supplementary Information

Supplementary Figure 1

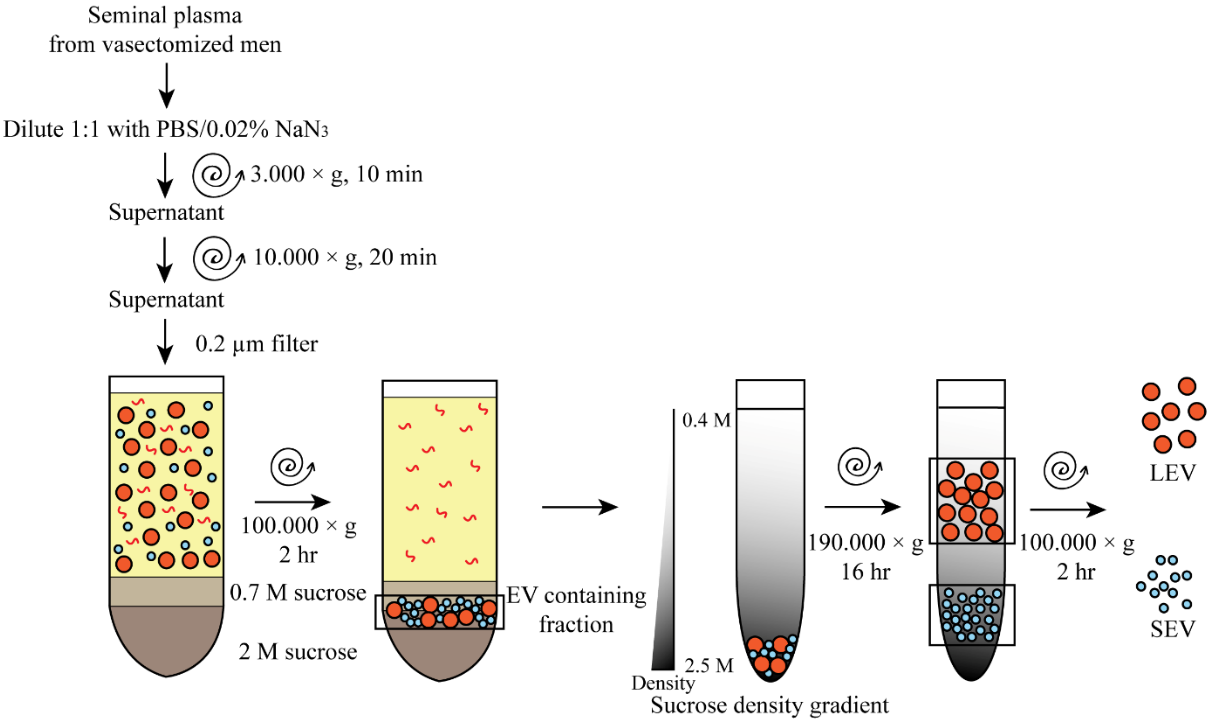

Supplementary Figure 1. Schematic overview of the method used to isolate LEV and SEV. Pooled samples of seminal plasma from vasectomized man was diluted 1:1 with PBS/0.02% NaN_3 and subjected to differential centrifugation up to 10,000 × g to remove cells, cell debris and majority of large microvesicles. The final supernatant was filtered through a 0.2 µm filter to ensure sterility. The samples were placed on top of a two layer sucrose block gradient and ultracentrifuged at 100,000 × g. EV were collected at the 0.7 M - 2 M sucrose interface. Solid sucrose was dissolved to increase its concentration to 2 M. The sample was overlayered with a linear sucrose density gradient (2 M to 0.4 M) and centrifuged for 16 h at 190,000 × g. Due to distinct velocities, LEV and SEV floated upwards into the gradient into different fractions.

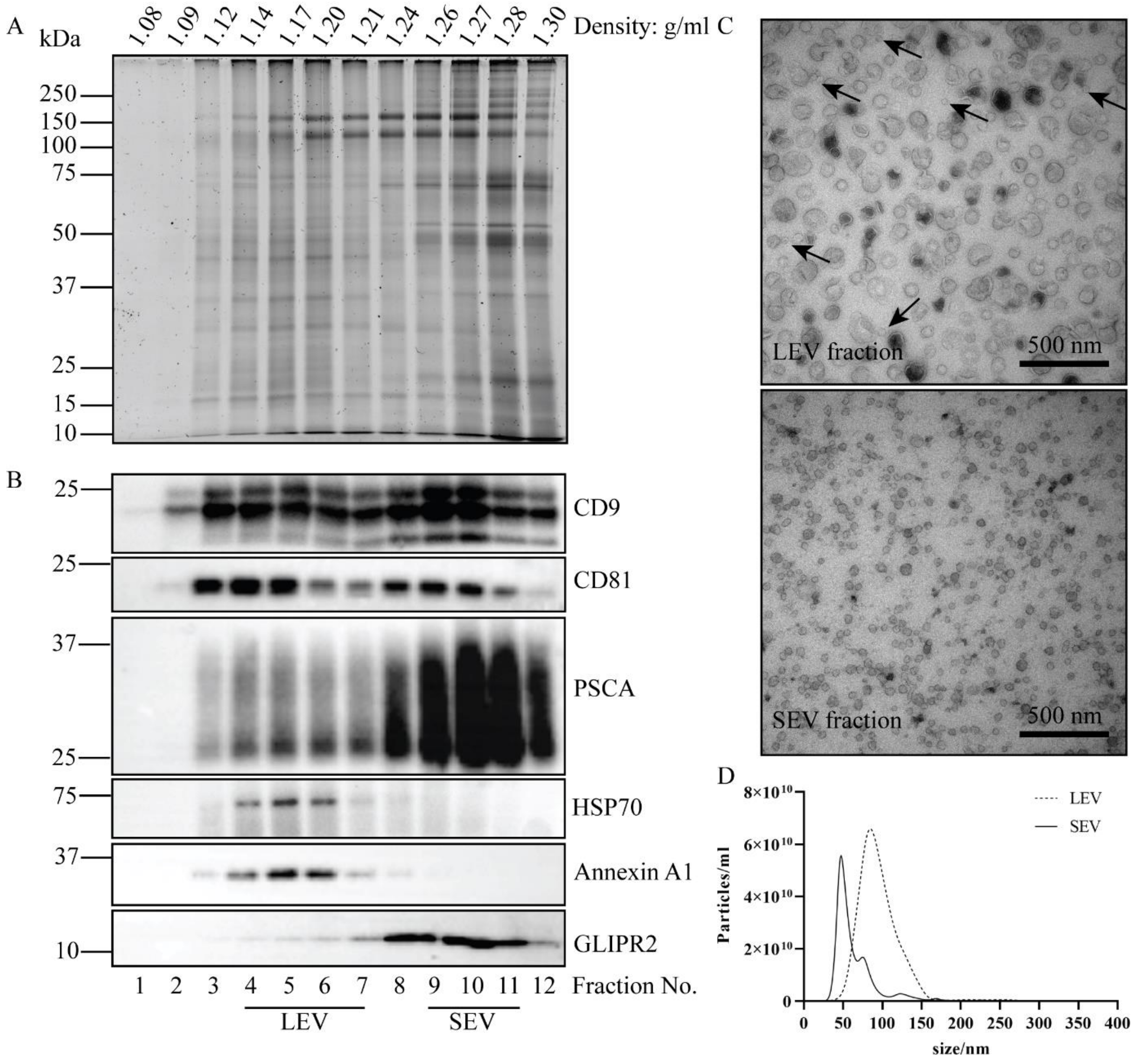

Supplementary Figure 2. Independent replicate of experiment in Figure 2.

Supplementary figure 3

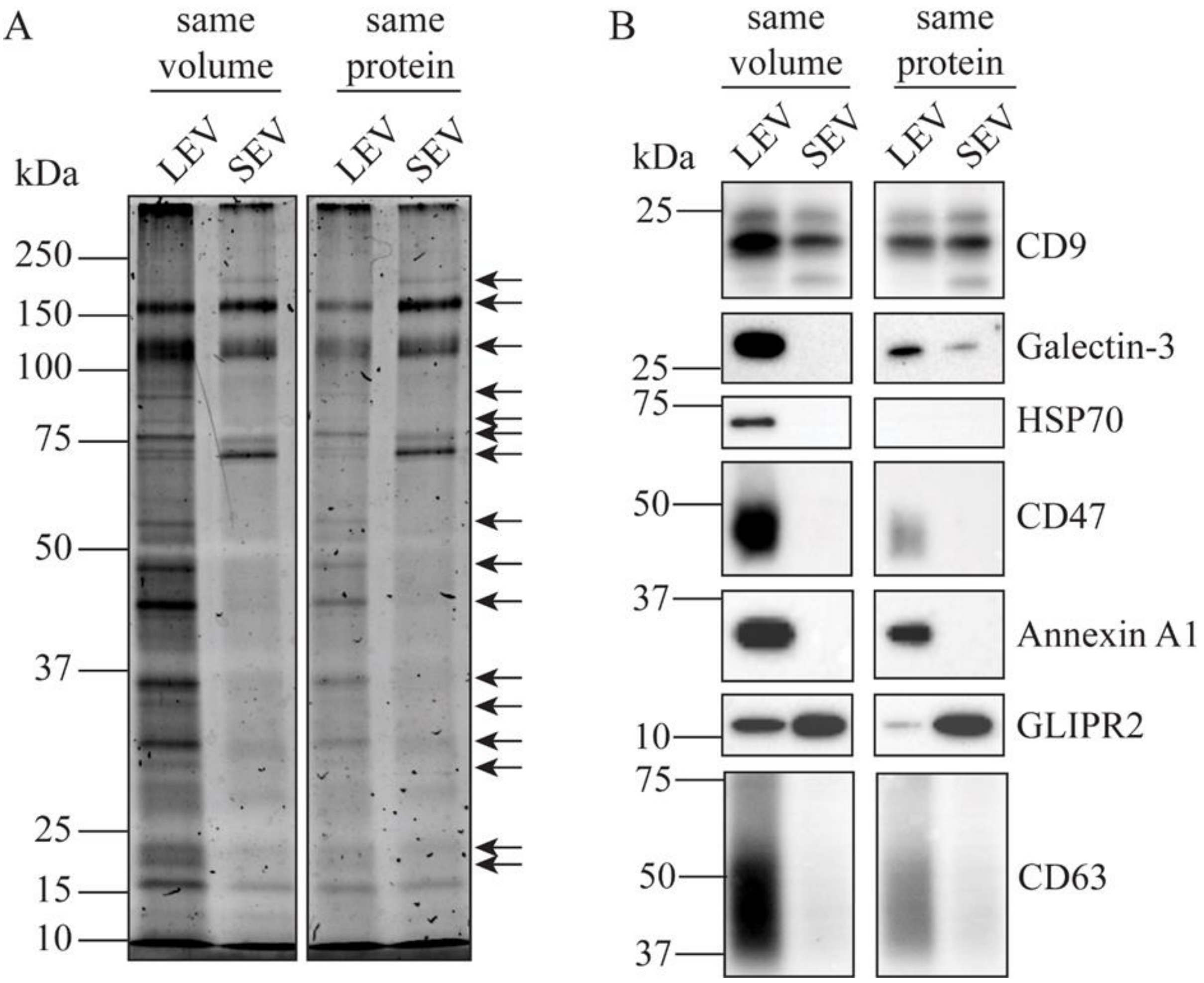

Supplementary Figure 3. Characterization of the LEV and SEV pooled sucrose density gradient fractions. LEV and SEV containing gradient fractions were determined as in Figure 1, and pooled. Pooled fractions were diluted with PBS and the EV collected by UC. (A), LEV and SEV isolates were analyzed by SDS-PAGE followed by Sypro ruby staining for total protein. Arrows indicate observed proteins differently present in LEV or SEV. (B), LEV and SEV isolates were analyzed by immunoblotting for the presence of EV associated proteins, including CD9, Galectin-3, HSP70, CD47, Annexin A1, GLIPR2, and CD63. Molecular weight markers are indicated on the left in kDa.
